# A comparison of various methods for measuring breast density and breast tissue composition in adolescent girls and women

**DOI:** 10.1038/s41598-022-17800-0

**Published:** 2022-08-08

**Authors:** Rebecca D. Kehm, E. Jane Walter, Ana Pereira, Melissa L. White, Sabine Oskar, Karin B. Michels, John A. Shepherd, Lothar Lilge, Mary Beth Terry

**Affiliations:** 1 Department of Epidemiology, Mailman School of Public Health, Columbia University, 722 W 168th St, New York, NY 10032 USA; 2 Princess Margaret Cancer Centre, University Health Network, 101 College St, Toronto, ON Canada; 3 Institute of Nutrition and Food Technology, University of Chile, Santiago, Chile; 4 Department of Epidemiology, Fielding School of Public Health, University of California, Los Angeles, 650 Charles E Young Dr S, Los Angeles, CA 90095 USA; 5 Faculty of Medicine and Medical Center, Insitute for Prevention and Cancer Epidemiology, University of Freiburg, Elsässerstr. 2, 79110 Freiburg, Germany; 6 Population Sciences in the Pacific Program, University of Hawaii Cancer Center, 701 Ilalo St, Honolulu, HI 96813 USA; 7 Department of Medical Biophysics, University of Toronto, 101 College St, Toronto, ON Canada; 8 Herbert Irving Comprehensive Cancer Center, Columbia University Medical Center, 1130 St Nicholas Ave, New York, NY 10032 USA

**Keywords:** Breast cancer, Cancer prevention, Cancer screening

## Abstract

This study compared different approaches to measuring breast density and breast tissue composition (BTC) in adolescent girls (n = 42, aged 14–16 years) and their mothers (n = 39, aged 36–61 years) from a cohort in Santiago, Chile. Optical spectroscopy (OS) was used to measure collagen, water, and lipid concentrations, which were combined into a percent breast density index (%BDI). A clinical dual-energy X-ray absorptiometry (DXA) system calibrated to measure breast density provided percent fibroglandular volume (%FGV) from manually delineated images. After digitizing mammogram films, the percent mammographic breast density (%MBD) was measured using computer-assisted software. Partial correlation coefficients (*r*_partial_) were used to evaluate associations between breast density measures and BTC from these three different measurement approaches, adjusting for age and body mass index. %BDI from OS was associated with %FGV from DXA in adolescent girls (*r*_partial_ = 0.46, *p*-value = 0.003), but not in mothers (*r*_partial_ = 0.17, *p*-value = 0.32). In mothers, %FGV from DXA was associated with %MBD from mammograms (*r*_partial_ = 0.60, *p*-value < 0.001). These findings suggest that data from OS, DXA, and mammograms provide related but distinct information about breast density and BTC. Future studies should explore how the information provided by these different devices can be used for breast cancer risk prediction in cohorts of adolescent girls and women.

## Introduction

Breast cancer susceptibility changes over time and is likely high during periods of changing breast tissue morphology and function, such as during adolescence^1,2^. Measuring breast density and other measures of breast tissue composition (BTC) across the lifecourse, particularly during windows of increased susceptibility, is thus essential for etiologic and prevention research. Mammographic breast density (MBD), which reflects the amount of fibrous and glandular tissue in the breast and can be measured on either the relative scale (%MBD) or absolute scale (absolute MBD, hereafter referred to as AMBD), is one of the strongest biomarkers of breast cancer risk^3–6^. The challenge is in evaluating breast density and other measures of BTC in adolescent girls and young women prior to the recommended age of screening mammography.

Two alternative approaches to measuring breast density and other measures of BTC are dual-energy X-ray absorptiometry (DXA) and optical spectroscopy (OS). DXA uses X-ray attenuation properties to directly measure dense and non-dense tissue and is commonly used to measure whole-body percent fat and body mass in pediatric studies because of its low radiation dose, high accuracy, and repeatability^7,8^. The DXA machine can be calibrated to measure breast density specifically. Previous studies support that breast density measurements from DXA and mammography are correlated, although the strength of the correlation differs for relative and absolute measurements (e.g., Spearman correlation coefficients: %FGV and %MBD = 0.76, absolute FGV (AFGV) and AMBD from craniocaudal and mediolateral views = 0.30 and 0.36, respectively)^9^. There are several advantages to using DXA instead of mammograms to measure breast density, including no breast compression, a much lower dose of radiation (a few days of background radiation per scan), and higher precision^7,8^. By contrast, OS is a non-imaging method, involving no breast compression or ionizing radiation, that measures the breast’s structural and metabolic bulk tissue properties by capturing the unique red and near-infrared light absorption and scattering properties of the different tissue components^10,11^.

OS provides information on major components of tissue composition, including water, collagen, and lipid, which can be combined to assess overall breast density. The ability of OS to measure individual chromophores, including collagen content apart from other fibroglandular tissue and hemoglobin content, represents a novel feature when considering potential biomarkers of breast cancer risk. This is because collagen content may promote epithelial cell proliferation and increase tumor mobility/invasion^12–14^. Hemoglobin content is associated with angiogenesis, and average oxygen saturation in the vasculature is an indicator of well-balanced oxygen supply and consumption^15^. Two light scattering parameters (amplitude and power) can also be obtained from OS, providing information on tissue structures at a microscopic level^16,17^. OS has been shown to identify women of mammographic screening age with > 75% MBD who are at elevated breast cancer risk^10,18,19^, and OS can detect breast development stages assessed by Tanner stage in adolescent girls^20^.

While both DXA and OS are promising methods for measuring breast density and BTC, particularly in cohorts of girls and young women prior to mammography screening age, no study has directly compared measures from these two devices. Thus, it not known if these devices provide equivalent or complementary information about breast tissue, making it difficult to compare findings across studies that have used these different techniques. This present study directly compared measures of breast density obtained on the same day from OS and X-ray attenuation where DXA was used in adolescent girls and both DXA and digital mammograms were used in adult women. Individual breast tissue chromophore concentrations and light scattering parameters from OS were also compared with the breast density measures from DXA and mammograms.

## Methods

### Study sample

A sample of 42 mother-daughter pairs were recruited from the Growth and Obesity Cohort Study at the Instituto de Nutrición y Tecnología de los Alimentos (INTA) at the Universidad de Chile. GOCS is an ongoing cohort of 1,190 Chilean children of low- and middle-income socioeconomic status (50% female), selected from the National Nursery School Council Program in the South East Area of Santiago, Chile. Children were between the ages of 2–4 years at initial study recruitment, which occurred in 2006^21^. For this comparison study, mother-daughter pairs were randomly selected who were available to participate in one clinic visit in May 2017 (21 pairs enrolled) or June 2018 (21 additional pairs enrolled). Trained research staff measured participants’ weight and height, which were used to calculate body mass index (BMI), and collected information on menstruation (e.g., date of menarche for daughters and menopausal status for mothers). DXA and OS exams were conducted on the same day at INTA, and same-day mammography appointments were arranged for participating mothers at another facility. All of the 42 daughters underwent OS and DXA exams on both breasts. All except one lactating mother underwent OS, DXA, and mammography exams on both breasts. The lactating mother was ineligible for DXA and mammography and thus only underwent OS; she was excluded from all analyses in this study. Written informed consent was obtained from mothers for her own and her daughter’s participation, along with informed assent from daughters. This study was approved by the Institutional Review Boards at Columbia University and the INTA. All methods in this study were conducted in accordance with relevant guidelines and regulations (Declaration of Helsinki).

### Dual-energy X-ray absorptiometry (DXA)

The DXA breast scanning protocol developed by one of the investigators (Shepherd; version 528) was used to measure fibroglandular volume (FGV) in mothers and daughters, as described in previous studies^22^. The imaging time was less than 30 s, and the radiation dose was approximately 15 µSv, or 15 µJ kg^-1^, per scan. This dose was approximately 10 times less than a standard screening mammogram and equivalent to about 2 days of background radiation. Each breast was scanned using the Prodigy DXA system software (version 13.6, series 200,675; GE Healthcare). A quality control phantom containing reference breast density materials was scanned throughout the study period to assure a stable calibration. DXA provides a true volumetric measure of breast density and has been shown to have high validity and precision for measuring breast density in girls throughout all Tanner stages^8,22^. The total projected breast area was manually delineated on each image allowing for breast FGV and total volume to be measured using a two-compartment model of adipose and fibroglandular tissue with software developed by the investigators^7,22^. %FGV was defined as the ratio of FGV relative to the breast volume multiplied by 100. %FGV was highly correlated between left and right breasts (*r*_Pearson_ = 0.91 and 0.78 in daughters and mothers, respectively); the average over both breasts was used in analyses, consistent with prior studies^9^.

### Optical spectroscopy (OS)

BTC and breast density were measured by OS using the red and near-infrared light transmissions in the spectral range of 650 to 1060 nm at 6 source-detector distances. Up to 12 spectra were captured per breast, optically interrogating different but overlapping tissue volumes^23^. Details of instrumentation used to gather attenuation spectra, including light source wavelengths, source and detector positions, frame shape, and cup sizes selection, are described elsewhere^23^. Instrument throughput was validated using a standard silicone reference before and after each participant’s breast measurement. A fitting algorithm was developed to determine the breast composition by minimizing the difference between simulated and measured spectra. The simulated spectra were calculated using the known absorption spectra of the different tissue chromophores and look-up tables of expected detector signal values for different tissue absorption and scattering properties, generated using a Monte-Carlo light propagation simulation program (FullMonte)^24^. The fitting algorithm yields the concentration of five major chromophores (water, lipid, collagen, oxyhemoglobin, and deoxyhemoglobin), which determine the absorption values used to calculate the simulated spectra, and two light scattering properties reflective of the cellularity of breast tissues from the OS spectrum (scattering amplitude and scattering power), which give the scattering values used to calculate the simulated spectra. The fit parameters were constrained to within expected ranges based on previous studies with generous latitude to allow for population differences and ensure physiologically reasonable values^25^. The sum of all chromophore concentrations was restricted to less than 100% (the five chromophore concentrations do not add up to 100% because there are other chromophores, such as myoglobin, in the breast tissue that have not been accounted for). Water, lipid, and collagen were constrained to 4–90%, 10–95%, and 1–30%, respectively; total hemoglobin (oxy and deoxy; hereafter referred to as hemoglobin) was constrained to 0.2–1% (4.9–23 µM); scattering amplitude was constrained to 0.1–4 mm^-1^ and scattering power to 0.05–5. Fitting was performed in MATLAB (The MathWorks Inc., Natick, MA, USA).

Only spectra with data for at least 7 wavelengths, matching the 7 unknown chromophore and scattering parameters, were used for the chromophore fitting (804 of 2,016 spectra with 7 wavelengths). To distinguish the primary lipid and water absorption peak, only spectra with usable data at 985 nm and data at either 905 or 940 nm were considered for subsequent data analysis. Applying these restrictions excluded 563 of the 804 spectra with 7 wavelengths, but there were still usable spectra data for all 42 daughters and 39 of the 41 mothers with DXA and mammography data. Chromophore fitting was performed for multiple starting points, and the best fit was selected based on the smallest least-squares difference between the measured and fitted spectra, with the additional constraint that no more than 2 parameters reached the predetermined minima or maxima. Chromophore and light scattering data were highly correlated between the left and right breasts (e.g., *r*_Pearson_ = 0.73 for the optical index, see below for details, between breasts). Therefore, data were averaged over all usable spectra from the multiple source-detector distances placed at different locations in both breasts to generate a single set of chromophore concentrations and light scattering properties for each participant. This is supported by previous research that has demonstrated symmetry in the optical spectra between breasts in the absence of breast disease^11^. Individual chromophores were combined to create a percent breast density index (%BDI), defined as [(collagen + water/collagen + water + lipid)*100], and an optical index, defined as [log((collagen + water)*scattering power)/lipid] and shown to significantly improve the confidence for assessing breast density compared with a single parameter^26^.

### Mammography

The craniocaudal and mediolateral oblique views were obtained from digital mammograms for both breasts using Hologic Selenia, Marlborough, MA, USA. Raw images were extracted from the mammogram machine and analyzed using VOLPARA® software (version 1.4.2; Matakina Technology, Wellington, New Zealand) to estimate the absolute dense volume and total breast volume on all 4 views. Only the mediolateral oblique views were used for this analysis, which are highly correlated to craniocaudal views^27^. %MBD was defined as the proportion of the dense volume of the breast relative to the total breast volume multiplied by 100 and averaged over both breasts (*r*_Pearson_ = 0.94 for %MBD in left and right breasts).

### Statistical analysis

The mean values of the breast density and BTC measures were compared in the sample of daughters (n = 42) and mothers (n = 39) using the two-sample t-test. Pearson correlation coefficients were calculated to compare breast density measures and BTC within daughter-mother pairs. Scatter plots were used to visually compare the relative and absolute measures of breast density from the different devices. Relative measures included %BDI from OS, %FGV from DXA, and %MBD from mammograms. Absolute measures of breast density from DXA (AFGV) and mammograms (AMBD) were compared with each other and with the summation of %collagen and %water (hereafter %collagen-water) from OS. Partial correlation coefficients were used to evaluate associations of the relative and absolute breast density measures from OS, DXA, and mammograms, adjusting for age and BMI. Menopausal status (mothers only) and age at menarche (daughters only) were also evaluated as covariates. However, adjustment for these covariates had little impact on the partial correlation coefficients, and thus these variables were not included in the final analysis for parsimony. Partial correlation coefficients, adjusted for age and BMI, were also used to evaluate associations of individual chromophore concentrations, light scattering parameters, and the optical index from OS with the relative measures of breast density from DXA and mammograms. This was done to determine if these measures provide additional information about BTC independent of the breast density measures provided by DXA and mammograms. All measures were standardized to a mean of zero and a standard deviation (SD) of one. Statistical significance was determined as *p*-value < 0.05 for a two-sided hypothesis test. Statistical analyses were conducted using Stata software version 15.1 (College Station, TX: Stata Corporation).

## Results

### Descriptive characteristics of daughters and mothers

At the time of the clinic visit for this study, daughters ranged in age from 14.0–16.3 years (mean ± SD = 15.1 ± 0.6), and mothers ranged in age from 36.9 to 61.2 years (mean ± SD = 47.5 ± 7.1); 64% of mothers were pre-menopausal at the study visit. Average BMI was 24.2 kg/m^2^ (SD = 5.1) and 30.7 kg/m^2^ (SD = 6.3) in daughters and mothers, respectively. Compared with mothers, daughters had, on average, higher percent breast density measured by OS (%BDI in daughters vs. mothers = 46.0 ± 9.8 vs. 38.9 ± 9.1, *p*-value = 0.001) and DXA (%FGV in daughters vs. mothers = 45.2 ± 15.4 vs. 31.6 ± 9.9, *p*-value < 0.001), but there was no difference in AFGV (Table 1). Several of the breast measures from OS were statistically significantly correlated in daughter-mother pairs, including %BDI (*r*_Pearson_ = 0.32, *p*-value = 0.048), optical index (*r*_Pearson_ = 0.50, *p*-value = 0.001), %lipid (*r*_Pearson_ = 0.47, *p*-value = 0.003), %hemoglobin (*r*_Pearson_ = 0.70, *p*-value < 0.001), and scattering power (*r*_Pearson_ = 0.41, *p*-value = 0.01). The DXA measures (%FGV and AFGV) were not significantly correlated in daughter-mother pairs.

**Table 1:** Comparison of breast tissue measures in daughters and mothers from the Growth and Obesity Cohort Study in Santiago, Chile.

Measure	Daughters (n = 42) Mean (SD)	Mothers (n = 39) Mean (SD)	T-test *P-*value^a^	Daughter-Mother Pairs Pearson correlations	
				*r*	*p-*value
**Relative measures of breast density**					
BDI from OS, %	46.0 (9.8)	38.9 (9.1)	0.001	0.32	0.048
FGV from DXA, %	45.2 (15.4)	31.6 (9.9)	< 0.001	− 0.06	0.73
MBD from mammograms, %	NA	7.3 (4.1)	NA	NA	NA
**Absolute measures of breast density**					
FGV from DXA, cm^3^	219.1 (78.9)	245.6 (100.1)	0.19	0.28	0.08
MBD from mammograms, cm^3^	NA	64.3 (23.6)	NA	NA	NA
**Additional measures of BTC from OS**					
Optical index	0.12 (0.68)	− 0.20 (0.95)	0.09	0.50	0.001
Lipid, %	46.0 (10.8)	52.6 (10.5)	0.01	0.47	0.003
Water, %	17.2 (4.8)	15.1 (7.3)	0.12	0.21	0.20
Collagen, %	21.3 (5.7)	17.9 (6.5)	0.02	0.12	0.48
Total hemoglobin, %	0.61 (0.16)	0.58 (0.15)	0.31	0.70	< 0.001
Scattering amplitude, mm-1	1.7 (0.5)	1.6 (0.5)	0.43	− 0.05	0.77
Scattering power	1.3 (0.5)	1.4 (0.8)	0.69	0.41	0.01

*BDI* breast density index, *BTC* breast tissue composition, *DXA* dual-energy X-ray absorptiometry, *FGV* fibroglandular volume, *MBD* mammographic breast density, *NA* not available, *OS* optical spectroscopy.

^a^*p*-value estimated from a two-sample t-test comparing sample means between daughters and mothers.

### Associations of relative measures of breast density from OS, DXA, and mammograms

**Figure 1:**
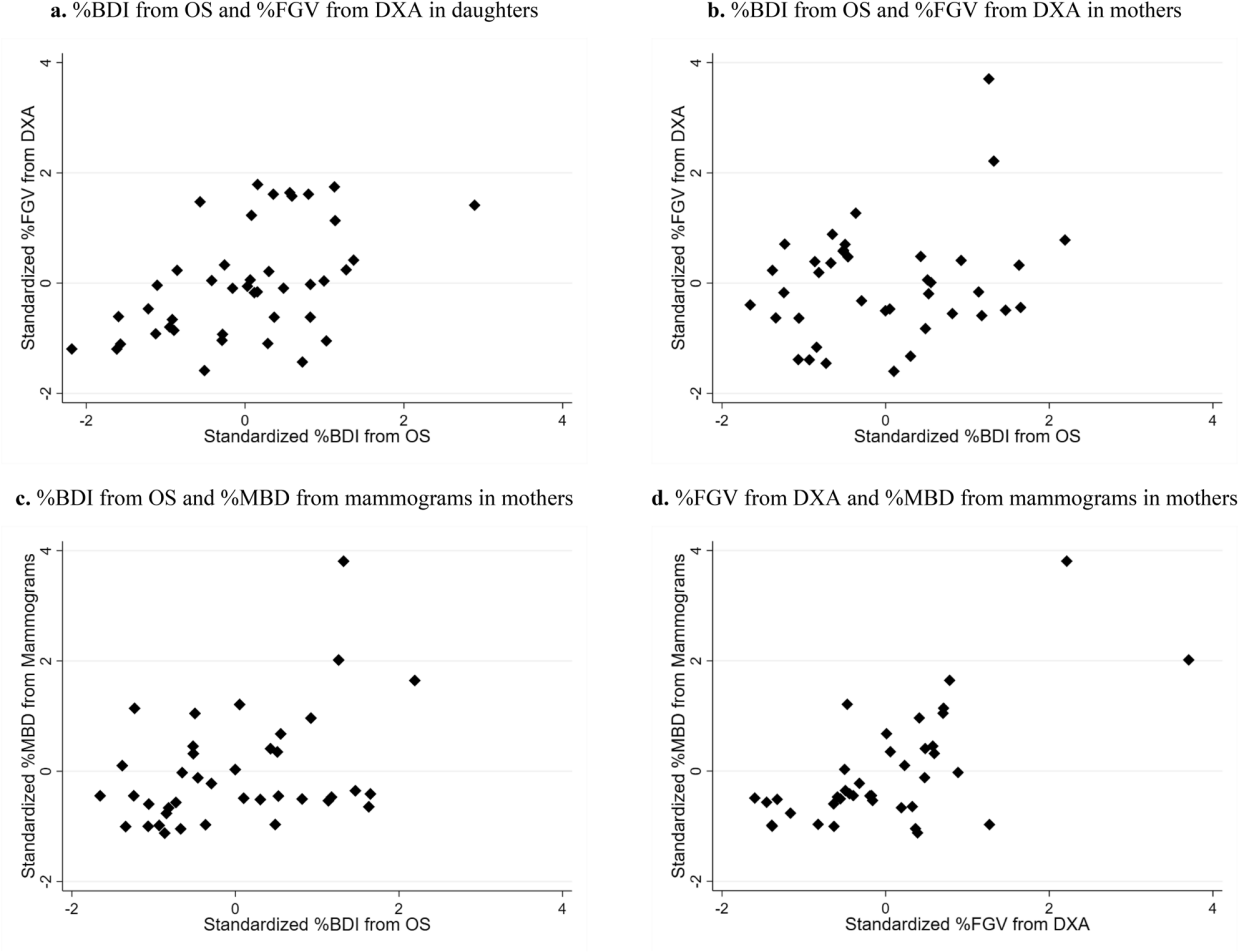
Scatter plots comparing relative measures of breast density from different devices in daughters and mothers from the Growth and Obesity Study in Santiago, Chile. Percent breast density index (%BDI) from optical spectroscopy (OS), percent fibroglandular volume (%FGV) from dual-energy X-ray absorptiometry (DXA) and percent mammographic breast density (%MBD) from mammograms are standardized to a mean of zero and a standard deviation of one. Observed values are presented as diamonds in scatter plots.

Scatter plots comparing the relative measures of breast density from the different devices are presented in Fig. 1. As shown in Table 2, after adjusting for age and BMI, there was a statistically significant positive association between %BDI from OS and %FGV from DXA in daughters (partial correlation coefficient (*r*_partial_) = 0.46, *p*-value = 0.003), as well as a statistically significant positive association between %FGV from DXA and %MBD from mammograms in mothers (*r*_partial_ = 0.60, *p* < 0.001). The association between %FGV and %MBD in mothers remained statistically significant after removing potential influential points (i.e., standardized values > 2).

**Table 2:** Partial correlation coefficients comparing breast density measures from different devices, adjusting for age and body mass index, in daughters and mothers from the Growth and Obesity Study in Santiago, Chile.

Measures of breast density compared between devices		Daughters (n = 42)		Mothers (n = 39)	
		*r*_partial_	*p*-value	*r*_partial_	*p*-value
**Relative measures**					
%BDI from OS	%FGV from DXA	0.46	0.003	0.17	0.32
%BDI from OS	%MBD from mammograms	NA	NA	0.25	0.13
%FGV from DXA	%MBD from mammograms	NA	NA	0.60	< 0.001
**Absolute measures**					
%Collagen-Water from OS	AFGV from DXA	0.31	0.051	0.15	0.39
%Collagen-Water from OS	AMBD from mammograms	NA	NA	0.15	0.37
AFGV from DXA	AMBD from mammograms	NA	NA	0.49	0.002

*AFGV* absolute fibroglandular volume, *AMBD* absolute mammographic breast density, *BDI* breast density index, *DXA* dual-energy X-ray absorptiometry, *NA* not available, *OS* optical spectroscopy.

### Associations of %collagen-water from OS with absolute breast density measures from DXA and mammograms

**Figure 2:**
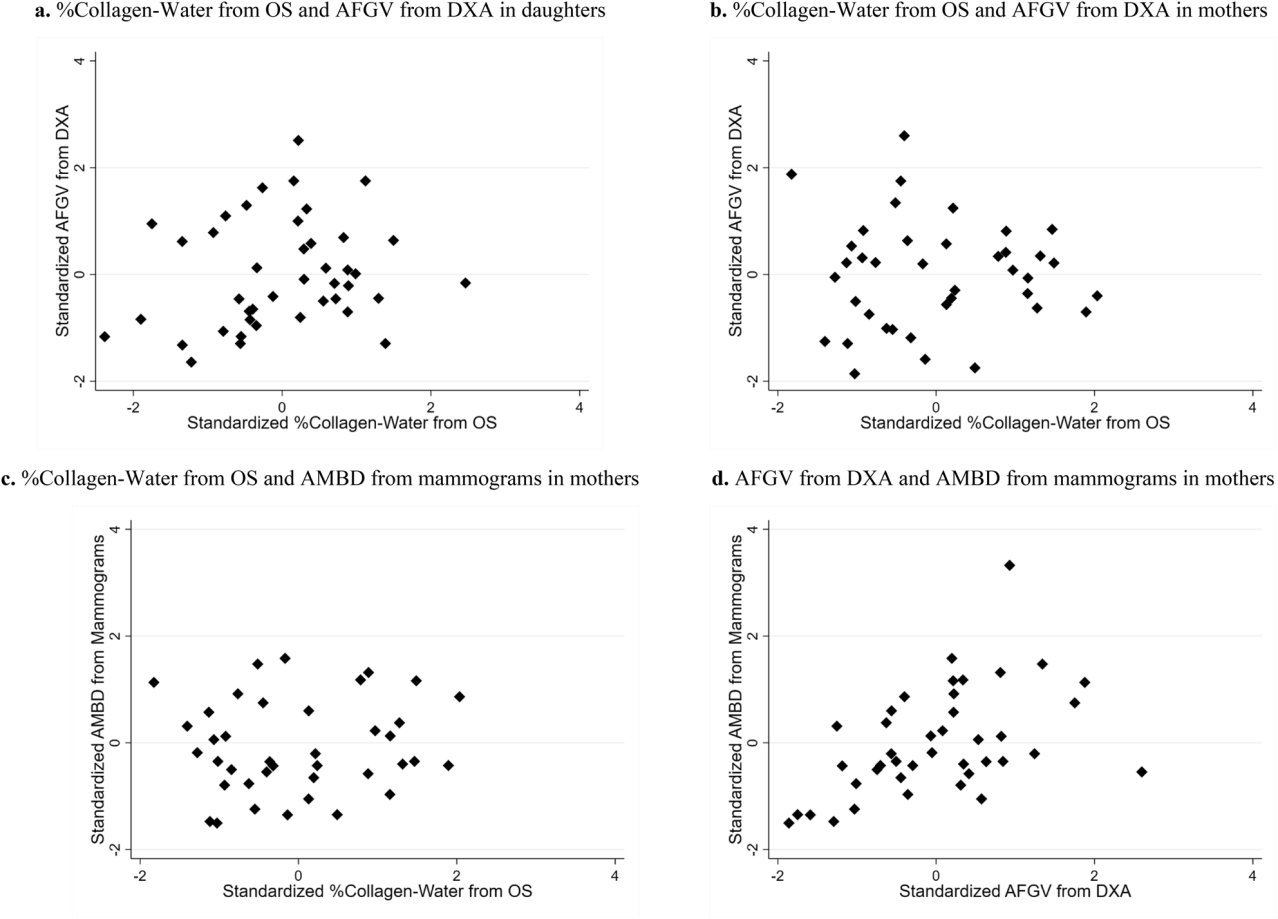
Scatter plots comparing absolute measures of breast density from different devices in daughters and mothers from the Growth and Obesity Study in Santiago, Chile. Percent collagen and water (%collagen-water, %C-W) from optical spectroscopy (OS), absolute fibroglandular volume (AFGV) from dual-energy X-ray absorptiometry (DXA), and absolute mammographic breast density (AMBD) from mammograms are standardized to a mean of zero and a standard deviation of one. Observed values are presented as diamonds in scatter plots.

Scatter plots comparing the absolute measures of breast density from the different devices are presented in Fig. 2. There was a positive association between %collagen-water from OS and AFGV from DXA in daughters after adjustment for age and BMI (*r*_partial_ = 0.31, *p*-value = 0.051). In mothers, AFGV from DXA was statistically significantly associated with AMBD from mammograms in mothers, adjusting for age and BMI (*r*_partial_ = 0.49, *p*-value = 0.002).

### Associations of additional measures of BTC from OS with relative breast density measures from DXA and mammograms

In daughters, the optical index (*r*_partial_ = 0.48, *p*-value = 0.002), %lipid (*r*_partial_ = -0.49, *p*-value = 0.001), and %collagen (*r*_partial_ = 0.33, *p*-value = 0.04) were each statistically significantly associated with %FGV from DXA, adjusting for age and BMI (Table 3). In mothers, only scattering amplitude (*r*_partial_ = 0.43, *p*-value = 0.01), which provides information on the overall density of particles at a microscopic level^16,17^, was associated with %FGV from DXA after adjusting for age and BMI. In contrast, the optical index (*r*_partial_ = 0.35, *p*-value = 0.04), scattering amplitude (*r*_partial_ = 0.52, *p*-value = 0.001), scattering power (*r*_partial_ = 0.47, *p*-value = 0.003), %water (*r*_partial_ = 0.36, *p*-value = 0.03), and %hemoglobin (*r*_partial_ = 0.37, *p*-value = 0.02) were each associated with %MBD from mammograms in mothers, adjusting for age and BMI.

**Table 3:** Partial correlation coefficients comparing breast tissue composition measures from OS with relative measures of breast density from DXA and mammograms, adjusting for age and body mass index, in daughters and mothers from the Growth and Obesity Study in Santiago, Chile.

Measure of BTC from OS	%FGV from DXA in daughters		%FGV from DXA in mothers		%MBD from mammograms in mothers	
	partial *r*	*p-*value	partial *r*	*p-*value	partial *r*	*p-*value
Optical index	0.48	0.002	0.05	0.79	0.35	0.04
Lipid, %	− 0.49	0.001	− 0.10	0.55	− 0.24	0.16
Water, %	0.17	0.29	0.21	0.22	0.36	0.03
Collagen, %	0.33	0.04	− 0.02	0.90	− 0.14	0.41
Total hemoglobin, %	0.18	0.28	0.08	0.63	0.37	0.02
Scattering amplitude, mm-1	0.10	0.52	0.43	0.01	0.52	0.001
Scattering power	0.25	0.11	0.13	0.44	0.47	0.003

*BTC* breast tissue composition, *DXA* dual-energy X-ray absorptiometry, *FGV* fibroglandular volume, *MBD* mammographic breast density, *OS* optical spectroscopy.

## Discussion

The dramatic increase in global breast cancer risk calls for new methods of measuring breast tissue characteristics. With the emergence and uptake of novel approaches to measuring breast density, comparison studies are needed to inform the validity of cross-study comparisons when these different techniques are used. Comparison studies are also needed to explore opportunities for data integration that can improve breast cancer risk prediction, particularly in younger cohorts. This is the first study to directly compare measures of breast density from OS and X-ray attenuation where DXA was used in adolescent girls and both DXA and digital mammograms were used in adult women. The relative and absolute measures of breast density from OS and DXA were associated in adolescent girls, although the association was stronger for the relative compared with absolute measures. In mothers, no associations were found between the breast density measures from OS and DXA. However, the relative and absolute breast density measures from DXA and mammograms were associated with each other, consistent with previous research^9^.

There are several reasons why breast density measured by OS might not be strongly correlated with the image-based breast density measures, particularly on an absolute scale. Unlike DXA and mammograms, which capture volumetric measures of dense and non-dense tissue for the entire breast, OS uses information averaged over various volumes between source-detector pairs strategically placed around the breast. In DXA and mammograms, the breast tissue volumes add equally to the overall measure, here FGV and MBD, respectively. In contrast, OS is not designed to evaluate overall tissue volume but rather the functional and physiological parameters of the breast, whereby the fraction of the interrogated breast volume is variable reaching a maximum of approximately 80% of the total breast volume. Volume elements close to the light sources and those close to the detectors are probed by more photons compared with breast tissue volume elements that are distal from the direct path between the sources and detectors. Additionally, while ionizing radiation used in DXA and mammography interacts with the atomic composition of the tissue, particularly with higher atomic number atoms (e.g., P, Ca), the optical photons used in OS interact with the electronic excitation of chromophores and variations in the tissue’s refractive index on a microscopic scale. Other factors such as DXA requiring manual segmentation of the total breast area could also contribute to differences in measurements across modalities.

When the individual chromophore concentrations and scattering parameters from OS were evaluated, some, but not all, of these measures were associated with the relative breast density measures from DXA and mammograms. Associations were in the expected direction based on what is known about the physiology of breast tissue. Specifically, the relative breast density measures from DXA and mammograms were anticipated to be negatively correlated with %lipid and positively correlated with %water and %collagen measured by OS. This is because %FGV and %MBD reflect the relative amount of fibroglandular tissue in the breast, comprised of connective and epithelial tissue rich in water and collagen, relative to the amount of adipose tissue that is high in lipid content. The positive associations between the scattering parameters from OS with %FGV and %MBD align with the fact that, optically, fatty tissues and the associated adipocytes are much larger and less structured compared with glandular cells. The light-scattering power of these larger cells is thus lower compared with connective tissues and cells associated with the glands^28^. Further, the positive association between %hemoglobin from OS and %MBD from mammograms is consistent with prior knowledge that increased vascularization and metabolism are expected in dense tissue, leading to higher total hemoglobin content^28,29^. Yet, partial correlations, adjusted for age and BMI, were all less than 0.50 when comparing measures from OS with DXA and mammograms, suggesting that OS provides information about BTC different from the breast density measures captured by DXA and mammograms.

Comparisons of breast measurements within daughter-mother pairs indicated that many of the breast measures from OS are correlated within pairs, suggesting that genetics and shared environmental factors may contribute to these breast tissue characteristics. However, other breast measurements from OS (water, collagen, and scattering amplitude), as well as the DXA measures, were not correlated in daughter-mother pairs, suggesting that independent factors such as age also impact breast density and BTC. It is well established that breast density is not static over the life course (e.g., increases during adolescence and decreases after menopause)^30^. Breast density measures from both OS and DXA were higher, on average, in daughters compared with mothers, which is consistent with previous research^31^. Partial correlations between DXA and OS measurements were also higher in daughters than mothers, which might be due to structural and functional changes in the breast over time that result in differences in the relative contributions of individual chromophores to the composite measure of breast density. For example, %collagen from OS was positively associated with %FGV from DXA in daughters but not mothers. Differential findings in daughters compared with mothers might also reflect the fact that daughters were more likely than mothers to require smaller cup sizes when using the OS device. The relative contributions of the signals from the pectoral muscle over the total breast tissue associated signal is higher in the small cup sizes compared with the larger cup sizes^32^.

This study is not without limitations. This includes the small sample size, which precluded more detailed analyses such as stratification by menopausal status in mothers or the categorization of breast density measures. Further, all girls were Tanner stage 4 + in our sample, so BTC at different stages of development could not be evaluated. Data on covariates such as exogenous hormone use and menstrual cycle timing were limited in this study, which might be important for understanding inter-group differences in BTC (e.g., differences between daughters and mothers)^33–35^. However, such factors do not need to be accounted for when comparing different device measurements that were taken on the same day in the same individual, which was the primary goal of this study. Therefore, despite these limitations, this study provides important comparative data on different modalities for measuring breast density and BTC in adolescent girls and women.

## Conclusions

This study supports that data from OS, DXA, and mammograms provide related but distinct information about breast density and BTC in adolescent girls and adult women. Comparisons between studies using these different devices should thus be made with caution, and more research is needed to understand how information from these different measurement techniques can be integrated to evaluate breast cancer risk. OS, in particular, might provide novel insights into structural and functional properties of the breast tissue across the lifecourse given that it does not involve radiation exposure, and can thus be used repeatedly starting at a young age, and provides data on BTC not captured by DXA or mammography (e.g., hemoglobin and collagen content). Yet, OS in its current state requires specialized equipment and personnel trained in operating the device and analyzing the data. Further device development and ease of use improvements are required to overcome these limiting factors to allow for widespread use of OS. The rapid development of new machine learning methods also has the potential to substantially increase the amount of information on BTC that can be extracted from image-based techniques. Optimally, DXA and OS or mammography and OS might be combined in research studies to provide more comprehensive data on breast density and BTC.

## Data Availability

The datasets used and analyzed during the current study are available from the corresponding author on reasonable request.
